# Claudin-4 Upregulation in Acantholytic and Autoimmune-Mediated Bullous Disorders

**DOI:** 10.3390/dermatopathology11010001

**Published:** 2023-12-21

**Authors:** Chau M. Bui, Huy G. Vuong, Minh-Khang Le, Kristin J. Rybski, Hatice B. Zengin, Haiming Tang, Bruce R. Smoller

**Affiliations:** 1 Department of Pathology and Laboratory Medicine, University of Rochester, Rochester, NY 14642, USA; kristin_rybski@urmc.rochester.edu (K.J.R.); hatice_zengin@urmc.rochester.edu (H.B.Z.); bruce_smoller@urmc.rochester.edu (B.R.S.); 2 Department of Pathology, University of Iowa, Iowa City, IA 52242, USA; huy-vuong@uiowa.edu; 3 Department of Pathology, University of Yamanashi, Yamanashi 409-3898, Japan; g20ddm26@yamanashi.ac.jp; 4 Department of Pathology, Yale School of Medicine, New Haven, CT 06510, USA; haiming.tang@yale.edu

**Keywords:** claudin-4, acantholytic disorders, autoimmune-mediated bullous disorders

## Abstract

Claudin-4 is a key component of tight junctions, which play an important role in the formation of the epidermal barrier by forming a circumferential network in the granular layer that serves as a gatekeeper of the paracellular pathway. The aim of this study is to illustrate claudin-4 immunohistochemical staining patterns of different blistering disorders. We collected 35 cases, including two Hailey–Hailey disease, one Darier disease, three Grover disease, one acantholytic acanthoma, two warty dyskeratoma, 11 pemphigus vulgaris (PV) including six mucosal PV, and two pemphigus foliaceus. For comparison, we included five cases of normal skin, five eczema, and three bullous pemphigoid cases. Claudin-4 demonstrated weak-to-moderate expression in keratinocytes located in the stratum granulosum, keratinocytes surrounding hair follicles, and adnexal glands. Further, claudin-4 exhibited moderate-to-strong membranous staining in disrupted keratinocytes surrounding and within the acantholytic and bullous areas in 16/22 of the acantholytic cases (not seen in the six cases of mucosal PV) and all three bullous pemphigoids. This finding suggests that claudin-4 is upregulated in these conditions, which may be a compensatory response to the disrupted barrier function. This finding could shed light on the molecular mechanisms underlying disrupted barrier function in blistering disorders, independent of the specific underlying disease mechanism.

## 1. Introduction

Acantholytic disorders (ADs) are disorders of the skin or mucosa characterized by the separation of keratinocytes at their desmosomal junctions. ADs encompass a diverse range of conditions, from the family of autoimmune pemphigus diseases, where autoantibodies directly damage desmosomes leading to keratinocyte separation and blister formation, to inherited disorders such as Darier disease and Hailey–Hailey disease, where mutations in calcium pump genes result in desmosomal instability [1]. These conditions, whether autoimmune-mediated or genetically inherited, can significantly impact the epidermal barrier function, compromise skin integrity, and potentially give rise to medical complications.

Several recent studies have begun to explore the molecular mechanisms underlying the pathogenesis of these disorders. Tight junctions (TJs) are a crucial component of the epidermal barrier, functioning to maintain intercellular integrity and to regulate paracellular permeability. The transmembrane protein claudin-4 has emerged as a central player in TJ regulation. It forms a circumferential network within keratinocytes that comprise the stratum granulosum, acting as a gatekeeper for the paracellular pathway [2].

The objective of this study was to characterize the immunohistochemical staining patterns of claudin-4 in various acantholytic disorders, namely, Hailey–Hailey disease, Darier disease, Grover disease, acantholytic acanthoma, warty dyskeratoma, pemphigus vulgaris (PV), and pemphigus foliaceus. By investigating claudin-4 expression across these diverse conditions, our aim is to shed light on its role in maintaining barrier function in the face of TJ damage sustained in these conditions.

## 2. Materials and Methods

This was a retrospective study. Formalin-fixed, paraffin-embedded, whole tissue sections from cases of Hailey–Hailey disease, Darier disease, Grover disease, acantholytic acanthoma, warty dyskeratoma, pemphigus vulgaris, and pemphigus foliaceus between 2015 and 2023 were retrieved from our surgical pathology archive. Cases of bullous pemphigoid, eczematous dermatoses, and unremarkable skin were included for comparison. Clinical information, hematoxylin and eosin-stained slides, and direct immunofluorescence studies of all cases were reviewed to confirm the diagnosis.

Sections were stained with immunohistochemistry for claudin-4 (EP417 clone, Rabbit monoclonal primary antibody, Predilute Ready-To-Use). The pattern of claudin-4 expression and the intensity of staining were tabulated for each case. Claudin-4 positivity was defined on the basis of membranous expression only. The staining intensity was graded as negative (0), weak (1+), moderate (2+), or strong (3+). Quantification of claudin-4 intensity was performed by two independent investigators in a blinded manner.

## 3. Results

We collected a total of 35 cases from patients aged between 30 and 87 years, comprising 18 males and 17 females. The cases encompassed a variety of conditions: two cases of Hailey–Hailey disease, one case of Darier disease, three cases of Grover disease, one case of acantholytic acanthoma, two cases of warty dyskeratoma, 11 cases of pemphigus vulgaris (including five cutaneous PV and six mucosal PV), two cases of pemphigus foliaceus, three cases of bullous pemphigoid, five cases of eczematous conditions, and five unremarkable skin cases (take from margins of surgical excisions).

Claudin-4 immunohistochemistry demonstrated weak-to-moderate expression in the keratinocytes located in the stratum granulosum, keratinocytes surrounding hair follicles, and adnexal glands (normal staining pattern) in all 35 cases.

**Figure 1:**
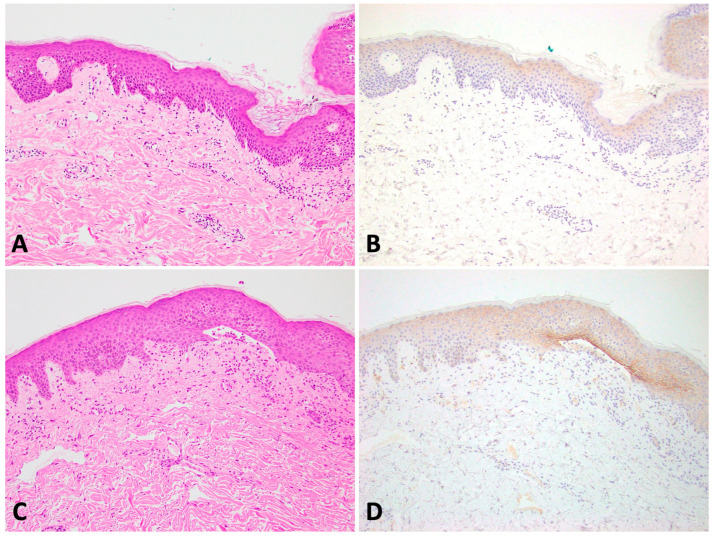
(**A**,**B**): Eczema with normal claudin-4 staining pattern. (**C**,**D**): Bullous pemphigoid (Left column—H&E 100×; Right column—Claudin-4 100×).

**Figure 2:**
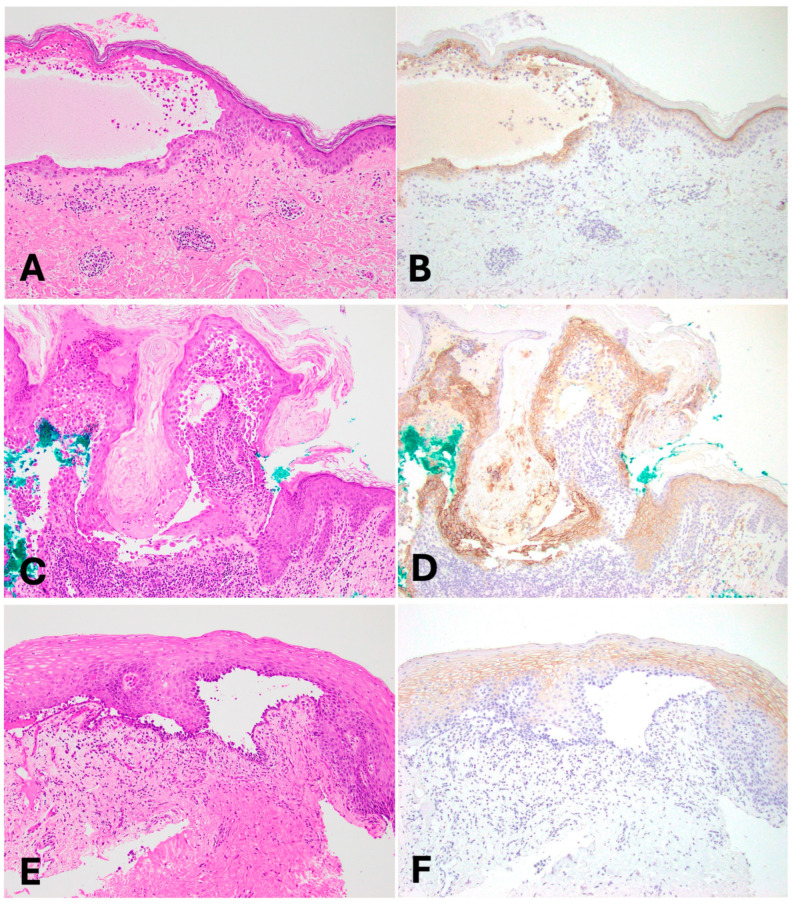
(**A**,**B**): Cutaneous pemphigus vulgaris. (**C**,**D**): Warty dyskeratoma. (**E**,**F**): Mucosal pemphigus vulgaris; (Left column—H&E 100×; Right column—Claudin-4 100×).

Furthermore, in all three cases of bullous pemphigoid and the 16/22 cases of epidermal acantholytic disorders—namely Hailey–Hailey disease, Darier disease, Grover disease, acantholytic acanthoma, warty dyskeratoma, cutaneous pemphigus vulgaris, as well as pemphigus foliaceus—there was moderate-to-strong membranous staining of claudin-4 in disrupted keratinocytes within acantholytic and/or bullous areas. This distinctive staining pattern displayed a tapering effect when transitioning away from the regions of disruption. It is important to note that this distinct staining pattern was not observed in any of the six cases of mucosal PV, five cases of eczema, or five cases of normal skin, as illustrated in Figure 1 and Figure 2. Notably, claudin-4 staining in eczema exhibited markedly weak to negative expression in lesional area (the stratum spinosum with spongiosis) compared with non-lesional skin (the stratum granulosum) where moderate staining was observed. In these cases of eczematous dermatitis, no disruption of cell–cell adhesions was apparent on routine histologic sections. The claudin-4 staining pattern in eczema is identical to that of normal skin. A summary of these findings is presented in Table 1.

## 4. Discussion

The stratum corneum of the epidermis plays a crucial role as the initial barrier preventing the entry of chemical, physical, or biological agents into the body. TJs are situated just beneath the stratum corneum and serve as a secondary barrier controlling the transit of small molecules, ions, and pathogens between neighboring cells while also preventing water loss [3,4]. Epidermal TJs are predominantly located in the granular cell layer and contribute to the formation of the stratum granulosum, but also extend across various other epidermal layers [5,6]. Beyond their barrier function, TJs also actively participate in processes such as proliferation, differentiation, cell–cell adhesion, and the apoptosis of keratinocytes [7,8].

Claudins represent a diverse family of integral membrane proteins that serve as critical components of TJs. These claudins exhibit specific expression patterns across various cells and tissues, contributing to the distinctive selective permeability characteristics of each tissue. Notably, claudin-1 and claudin-4 play key roles in the barrier functions of the skin, while claudin-2 and claudin-15 are linked to paracellular transport in the intestinal epithelium [9]. Claudin-4 displays a distinctive expression pattern within the stratum granulosum of the epidermis governed by ΔNp63, a transcription factor belonging to the p53 family [3]. Knockdown of claudin-4 in mice leads to early death due to severe transepidermal water loss, highlighting the crucial role of claudin-4 in maintaining efficient epidermal barrier function [10,11].

Previous research has highlighted the significance of TJ proteins, including claudin-4, in the pathophysiology of various skin diseases [12,13,14,15]. In this study, we have demonstrated that claudin-4 consistently exhibits weak-to-moderate expression in the keratinocytes of the stratum granulosum (as has been previously reported) [12,13], and the keratinocytes surrounding hair follicles and adnexal glands [15]. Claudin-4 expression was stronger within the keratinocytes located in areas affected by acantholysis or blistering. This enhanced expression contrasted sharply with the relatively lower levels found in the overlying granular layer and within the hair follicles. This observation held true across all types of acantholytic disorders and bullous pemphigoid disease studied, but not in control cases of eczematous dermatitis or unremarkable skin, nor in our cases of mucosal PV. This observation underscores the potential role of claudin-4 in these specific skin regions. In line with our findings, Raiko et al. found similar patterns of claudin-4 expression in acantholytic skin disorders such as Hailey–Hailey disease and Darier’s disease, revealing that claudin-4 localizes to the upper epidermis and acantholytic cells within Hailey–Hailey blisters and Darier’s disease [12]. Furthermore, a study by Li et al. demonstrated that the significant upregulation of claudin-4 might serve as a compensatory mechanism aimed at maintaining barrier function in affected skin regions [16]. This upregulation in claudin-4 may serve to counteract the barrier disruption caused by the downregulation of claudin-1 in these disorders [16].

Another study by Hatakeyama et al. investigating the expression patterns of adhesion molecules in the gingival mucosa reported the presence of claudin-1 in the intermediate portion of the uppermost epidermal layers, but only in a few cases. The authors proposed that TJs in the oral mucosa could resemble structures present in the uppermost epidermal layer, akin to the stratum granulosum in the skin epidermis [17]. Contrasting this, our own study revealed a distinctive difference in claudin-4 expression. We noted the presence of claudin-4 exclusively within disrupted keratinocytes in acantholytic and bullous areas of cutaneous PV, whereas mucosal PV exhibited no such claudin-4 presence. These observations suggest the potential absence (or markedly diminished expression) of claudin-4 in the mucosa and the possibility that the EP417 antibody used for claudin-4 immunohistochemistry may not cross-reactively stain claudin-1. In a more recent study, it was revealed that the baseline expression of TJ genes differs between oral and skin epithelium, and these expression patterns change during wound healing. Notably, the knockdown of claudin-1 and occludin resulted in alterations in the proliferation and migration in skin epithelium, while no such effects were observed in oral keratinocytes [18]. In the skin epithelium, claudin-1 knockdown triggers claudin-4 overexpression, possibly compensating for the loss. However, in the oral mucosal epithelium, claudin-1 knockdown leads to reduced keratinocyte proliferation and migration, preventing claudin-4 overexpression [18]. These findings offer an explanation for our study’s results. Collectively, these observations emphasize the multifaceted role of claudins in maintaining barrier function in different sites and across different diseases.

Significantly, claudin-4 staining in all eczema cases in our study revealed stronger expression in non-lesional skin (the stratum granulosum) than in lesional skin, aligning with the pattern identified in the 2015 study by Gruber et al. [15]. This observation implies an upregulation of Claudin-4 in non-lesional areas, highlighting a distinctive contrast in expression between non-lesional and lesional eczematous skin. A noteworthy contribution of our study is the first-time report of claudin-4 overexpression in the keratinocytes surrounding blisters in an autoimmune-mediated subepidermal bullous disorder, bullous pemphigoid. The underlying pathogenesis of this observation remains unclear and warrants further exploration. It is crucial to acknowledge the limitations of our study, including the relatively small size of our current cohort and the utilization of a single antibody marker. To validate and generalize our findings, further extensive studies involving larger cohorts and multiple antibody markers are imperative.

## 5. Conclusions

In summary, our study has revealed that claudin-4 displays distinct expression patterns in both acantholytic and autoimmune-mediated bullous skin diseases. This discovery contributes to our understanding of the role of claudin-4 in these conditions and provides valuable insights into the intricate molecular mechanisms that contribute to the compromised barrier function observed in these skin conditions. Ultimately, this knowledge could serve as a foundation for the development of novel therapeutic approaches aimed at mitigating the breakdown in barrier function observed in these disorders.

## Figures and Tables

**Table 1:** Summary of claudin-4 staining pattern in all cases.

Entity	Number of Cases	Normal Claudin-4 Staining Pattern	Claudin-4 Overexpression in Disrupted Keratinocytes within Acantholytic and/or Bullous Areas
Hailey–Hailey disease	2	1+	2+
Darier disease	1	1+	3+
Grover disease	3	1+	3+
Acantholytic acanthoma	1	1+	2+
Warty dyskeratoma	2	1+	3+
Pemphigus foliaceus	2	1+	3+
Cutaneous Pemphigus vulgaris	5	2+	3+
Mucosal Pemphigus vulgaris	6	2+	0
Bullous pemphigoid	3	1+	3+
Eczema (spongiotic dermatitis)	5	2+	NA
Normal skin	5	1+	NA

NA: Not applicable.

## Data Availability

Data are contained within the article. The data presented in this study are available on request from the corresponding author.

## References

[1] Dhitavat J., Fairclough R.J., Hovnanian A., Burge S.M. (2004). Calcium pumps and keratinocytes: Lessons from Darier’s disease and Hailey-Hailey disease. Br. J. Dermatol..

[2] Schneeberger E.E., Lynch R.D. (2004). The tight junction: A multifunctional complex. Am. J. Physiol. Cell Physiol..

[3] Kubo T., Sugimoto K., Kojima T., Sawada N., Sato N., Ichimiya S. (2014). Tight junction protein claudin-4 is modulated via Δnp63 in human keratinocytes. Biochem. Biophys. Res. Commun..

[4] Ozawa T., Sugawara K., Tsuruta D. (2014). The discovery of epidermal tight junctions. Exp. Dermatol..

[5] Kubo A., Nagao K., Yokouchi M., Sasaki H., Amagai M. (2009). External antigen uptake by Langerhans cells with reorganization of epidermal tight junction barriers. J. Exp. Med..

[6] Ishida-Yamamoto A., Kishibe M., Murakami M., Honma M., Takahashi H., Iizuka H. (2012). Lamellar granule secretion starts before the establishment of tight junction barrier for paracellular tracers in mammalian epidermis. PLoS ONE.

[7] O’Neill C.A., Garrod D. (2011). Tight junction proteins and the epidermis. Exp. Dermatol..

[8] De Benedetto A., Rafaels N.M., McGirt L.Y., Ivanov A.I., Georas S.N., Cheadle C., Berger A.E., Zhang K., Vidyasagar S., Yoshida T. (2011). Tight junction defects in patients with atopic dermatitis. J. Allergy Clin. Immunol..

[9] Chiba H., Osanai M., Murata M., Kojima T., Sawada N. (2008). Transmembrane proteins of tight junctions. Biochim. Biophys. Acta Biomembr..

[10] Furuse M., Hata M., Furuse K., Yoshida Y., Haratake A., Sugitani Y., Noda T., Kubo A., Tsukita S. (2002). Claudin-based tight junctions are crucial for the mammalian epidermal barrier: A lesson from claudin-1-deficient mice. J. Cell Biol..

[11] Kirschner N., Rosenthal R., Furuse M., Moll I., Fromm M., Brandner J.M. (2013). Contribution of tight junction proteins to ion, macromolecule, and water barrier in keratinocytes. J. Investig. Dermatol..

[12] Li J., Li Q., Geng S. (2019). All-trans retinoic acid alters the expression of the tight junction proteins Claudin-1 and -4 and epidermal barrier function-associated genes in the epidermis. Int. J. Mol. Med..

[13] Raiko L., Leinonen P., Hägg P., Peltonen J., Oikarinen A., Peltonen S. (2009). Tight junctions in Hailey-Hailey and Darier’s diseases. Dermatol. Rep..

[14] Bui C.M., Balzer B.L., Shon W. (2023). Claudin-4 Expression in the Distinction of Mammary and Extramammary Paget Disease from Morphologic Mimics: Diagnostic Utility and Pitfalls. Am. J. Dermatopathol..

[15] Hatakeyama S., Ishida K., Takeda Y. (2010). Changes in cell characteristics due to retinoic acid; specifically, a decrease in the expression of claudin-1 and increase in claudin-4 within tight junctions in stratified oral keratinocytes. J. Periodontal. Res..

[16] Leonardo T.R., Shi J., Chen D., Trivedi H.M., Chen L. (2020). Differential expression and function of bicellular tight junctions in skin and oral wound healing. Int. J. Mol. Sci..

[17] Peltonen S., Riehokainen J., Pummi K., Peltonen J. (2007). Tight junction components occludin, ZO-1, and claudin-1, -4 and -5 in active and healing psoriasis. Br. J. Dermatol..

[18] Gruber R., Börnchen C., Rose K., Daubmann A., Volksdorf T., Wladykowski E., Vidal-Y-Sy S., Peters E.M., Danso M., Bouwstra J.A. (2015). Diverse regulation of claudin-1 and claudin-4 in atopic dermatitis. Am. J. Pathol..

